# Supplementary material for: No clear evidence of a difference between individuals who self-report an absence of auditory imagery and typical imagers on auditory imagery tasks
Source: PLoS One. 2024 Apr 3;19(4):e0300219. doi: 10.1371/journal.pone.0300219 (PMC10990234; doi:10.1371/journal.pone.0300219)
Supplement: Attachment — Submitted filename: R2_ResponseToReviewers.docx [file pone.0300219.s002.docx]

Please find a revised version of our manuscript*.* We thank the reviewers for their reviews of our manuscript. We have highlighted the changes that have been made in the manuscript and provide a response to each comment below subsequent to this round of review.

**Response to reviewer comments**

Reviewer #1: The authors fully addressed most of my previous comments.

**The only point I think could due with some additional discussion is the correlations between the questionnaires and the auditory pitch task. If I understood this correctly, these two measurements are positively correlated in the control group – suggesting that subjective reports of vividness correlate with objective performance in this task – but there is no difference between the aphantasic and control groups, which are themselves defined based on the subjective reports (low VVIQ versus high VVIQ).**

**The authors suggest that this might be because ‘aphantasic participants were more greatly motivated to perform well on this task given their self-reported difficulties, which acted to compensate for their imagery deficits.’ But this seems a little far-fetched to me. Are other explanations possible? For example, perhaps aphantasic participants use different strategies to perform the same task (e.g. as suggested by Koegh, Wicken & Pearson, 2021 for working memory). Could the authors speculate more on this?**

We agree that this is unlikely, but we were asked to speculate on whether ‘demand characteristics’ could have influenced our findings in the previous round of review. Given that we are reporting null results, this was one way that “demand characteristics” could have influenced the pattern of results, under the expectation that aphantasics should ordinarily perform worse than control participants. We have changed the emphasis and incorporated your suggestion, it now reads:

*“Given that this task explicitly asked aphantasic participants to generate auditory imagery, it may have been subject to ‘demand characteristics’. However, one might assume that such a bias would lead to reduced rather than increased performance in the aphantasic group. Given that performance was equivalent between the groups, it may be that participants with aphantasia drew upon alternative non-imagery-based strategies to complete the tasks”*

Reviewer #2: *** beginning of review

General issues:

**Relationship of Aphantasia to Perception. The authors address the possibility of aphantasia across different modalities in imagery. Even so, I found myself wondering (e.g., while reading lines 70-83) about the relationship between aphantasia in a given modality and perceptual functioning within that same modality. Given that there is a correlation between patterns of brain activation during perception in a given modality and imagery in that same modality, one suspects aphantasia might be related to deficits in perception. This would also seem related to the question of whether imagery draws on a single unitary mechanism or multiple cognitive mechanisms (cf. line 83). Although the relationship between imagery and perception is briefly addressed on lines 143-149, perhaps this might be moved to earlier in the introduction and expanded. Indeed, if auditory aphantasia is not linked to any auditory perceptual deficits, then that would have significant implications for understanding such aphantasia, as well as understanding of imagery more generally.**

We thank the reviewer for this suggestion. However, we feel that this point is already adequately expressed in lines 143-149 (as pointed out by the reviewer). We would prefer not to change the structure of the introduction of our manuscript to accommodate this request. We feel that this point is already clearly made and as such do not want to change the existing structure and organisation of the arguments in the introduction section. We leave it to the action editor to decide whether this change is required.

**Specifying the Modality. There are multiple instances in which in the authors refer to “mental images”. Sometimes it is not clear if they are referring to both visual images, auditory images, or both (unimodal) visual and auditory images (e.g., lines 419, 421, 448, etc.). Relatedly, visual imagery and auditory imagery are treated as separate types of unimodal images, but would the same patterns of aphantasia occur if a single multimodal image were generated? Also, I would suggest that rather than the general “mental imagery” the authors always use the more specific “visual mental imagery” or “auditory mental imagery” whenever possible. There are also a few instances in which the authors refer to “aphantasia participants”, but it isn’t clear whether the aphantasia being referred to is visual or auditory (e.g., line 533).**

Where appropriate, we have made sure that we have specified if the imagery in reference is visual or auditory. When we discuss imagery ‘in general’ we refer to imagery without mention of the specific modality of the imagery.

**“Control Group” vs. “Typical Imagery Group”. The authors seem to alternative between referring to their control group as the “control group” (e.g., lines 388, 390, 413, 458, etc.) and referring to their control group as the “typical imagery group” (e.g., lines 448, 453, 456, 476, etc.). Using multiple terms for the same construct or group makes more work for the reader, and so the authors should just pick a single term and use that term more consistently.**

We now consistently refer to the ‘typical imagery control group’ to reduce the use of multiple terms.

Specific Issues:

**Lines 35-36: Line 35 states “it is possible to generate many different kinds of internal representation”, which implies generation of non-imagery forms of representation as well as generation of imagery, but all the examples listed on line 36 seem to involve imagery.**

We have changed this to refer to imagery experiences only.

**Lines 61-62: “are well documented” might be a bit strong. Perhaps “are relatively well documented” might be better.**

This has been changed.

**Line 78: Perhaps “in those domains” should be inserted after “require imagery”.**

This has been added.

**Line 105: What are “frank differences” and how do they differ from just “differences”? If there is a difference, it should be explained, and if there is no difference, perhaps “frank” should be deleted. Relatedly, what is a “frank deficit” (on line 168), and how does that differ from just a “deficit”? If there is a difference, it should be explained, and if there is no difference, perhaps “frank” should be deleted.**

The word ‘frank’ has been removed from both of these sentences.

**Lines 106-110: The statement “it is not clear… one explanation could be…” seems disorganized. More specifically, it seems strange to me that the authors start the second sentence with “one explanation…” given that they just spend lines 90-105 discussing other possible explanations for why differences were not more apparent. (e.g., imagery is epiphenomenal, qualitatively different or weaker imagery, etc.). Rather than making line 106 sound like a shift to a different topic (an explanation rather than a description), it might be better to make it clearer that additional explanations will be given.**

We have added to the end of the first sentence that there are several possible explanations, and changed the beginning of the following sentence (that begun “One explanation”) to “For example”. For clarity, this reads:

*“It is not clear why differences in performance on imagery tasks are not more apparent in aphantasic groups, however, there are several possible explanations. For example, One explanation could be that people with aphantasia use compensatory…”*

**Lines 130-134: A greater number of beats in an auditory image and a longer distance in a visual image are stimulus-specific properties of the images, and it isn’t entirely clear why a common mechanism would respond to these different types of stimulus-specific properties, and so perhaps some speculation on this might be offered.**

We have added an additional example of mental rotation to support this argument and we have also added an additional sentence that speculates at a common mechanism. This reads:

*For example, it takes longer to scan through an imagined melody that contains a longer series of beats (Halpern, 1988; Zatorre & Halpern, 2005) in a manner analogous to the increased time that it takes to visually scan imagined objects that are further apart in distance (Kosslyn, 1981; Pearson et al., 2015) or rotate objects that require larger angular rotations (Shepard & Metzler, 1971). This suggests that both auditory and visual imagery may be underpinned by common spatial mechanisms that allow for auditory images to be extended in time akin to the way visual images are extended in space.*

**Also, for consistency “ones” in line 130 should be replaced with “images”.**

This has been changed.

**Line 140: For clarity, I would add “the amount of” before “grey”**

This has been added.

**Lines 173-181: Was the VVIQ score used in recruiting participants, or was it only obtained after recruitment? The text implies the former, but I suspect the latter actually occurred. Regardless, this should be clarified. Also, did the forums from which the authors recruited participants distinguish between visual or auditory aphantasia? More specifically, did the participants actually self-identify as aphantastic based on their visual imagery, or might some self-identify as aphantastic based on their auditory imagery? Also, would not have recruiting on the basis of auditory aphantasia (rather than recruiting based on visual aphantasia) have also allowed examination of the relationship between visual aphantasia and auditory aphantasia? Other than possibly having more total respondents if they advertised for visual aphantasia, why didn’t the authors recruit from the population that they were really interested in, which was auditory aphantastics?**

The VVIQ was obtained following recruitment, we have referred to each groups’ VVIQ score following the descriptions of how the two groups were recruited.

There were theoretical and practical reasons for conducting recruitment in the way that we did.

This study was run from September 2019- March 2020, during this time, aphantasia was defined as an inability to generate visual imagery (Dawes et al., 2020; Zeman et al., 2015, Zeman et al., 2020), and more recent definitions have extended this definition to include the absence of other sensory modalities (Monzel et al., 2022), with terms such as “auditory aphantasia” or “visual aphantasia” commonly used to describe the absence of imagery within specific sensory modalities. We have added to the text the time frame of when this data was collected, as this is important in the context of aphantasia as the definition of aphantasia has altered within the last year. Our approach was motivated by the desire to understand the extent of the co-occurrence of auditory and visual imagery deficits in people that call themselves “aphantasic”.

Aphantasia is a niche population (experienced by less than 5% of the population). It is difficult to recruit for studies of people with aphantasia for this reason. Thanks to a growing recognition of the condition we are lucky to be able to recruit via various aphantasia interest groups. These groups do not currently exist for people with “auditory imagery deficits”.

We have added the following to acknowledge the shift in aphantasia definitions and contextualise our study in relation to these studies prior to this definition shift:

*“This study was undertaken between September 2019- March 2020, during which, aphantasia was defined specifically as the absence of visual imagery (Zeman et al., 2015; Zeman et al., 2020) rather than an absence of sensory imagery (Monzel et al., 2022). We recruited participants on this basis to investigate the association of auditory and visual imagery deficits in individuals that would typically identify as being aphantasic (on the basis of their reduced visual imagery). This approach was taken to investigate the auditory imagery of individuals that would typically be identified as ‘aphantasic’ in previous research studies in this area that were collected during this time.”*

We also draw attention to the fact that we explicitly discuss whether this recruitment strategy may have influenced the findings in our discussion.

**Line 176, 217, 298: I think using the Oxford comma usually improves clarity, and so I would insert a comma after “Twitter” on line 176, after “preferences” on line 217, and after “emotion” on line 298. Of course, if I missed any lists of three or more items that didn’t already include an Oxford comma, then analogous comments would apply to those lists.**

We have added these commas and added them where appropriate.

**Lines 209-212: I realize the data for the control subscale will eventually be discarded, but this subscale should still be fully described. An example question from the subscale should be given and the types of transformations described. Also, anchor terms for both the vividness subscale and the control subscale should be provided.**

We have amended this description of the BAIS-C subscale and provided the anchor descriptions for both scales. The BAIS description reads as follows:

*“The BAIS (Halpern, 2015) is an auditory imagery questionnaire that comprises two scales: the vividness (BAIS-V) and control (BAIS-C) subscales. The vividness subscale requires participants to reflect on the vividness with which they can imagine 14 sounds described in a written scenario (e.g. “consider attending a choir rehearsal, the sound of an all children’s choir singing the first verse of a song”) on a scale of 1 ("no image present at all”) to 7 (“as vivid as actual sound”). These scenarios involve imagining different kinds of sounds that include voices and musical instruments. The control subscale comprises of 14 pairs of imagined sounds, which require participants to rate ‘the ease of change’ or transition on a scale of 1 (“no image present at all”) to 7 (“extremely easy to change the image”) between two imagined auditory scenarios (e.g. “Consider listening to a rain storm. The sound of gentle rain. The gentle rain turns into a violent thunderstorm”).”*

**Line 218: A space should be inserted after “behaviour”.**

This space has been added.

**Lines 228-232: The authors refer to the General Musical Sophistication Scale as one part of the Goldsmiths Musical Sophistication Index, but when they describe scoring of the Scale, the claim “these values are summed to provide an overall score for the participants” sounds as if they are describing the Index (the Gold-MSI) rather than a separate General Musical Sophistication Scale. This needs to be clarified.**

We have clarified this to ensure we are referring to the General Musical Sophistication Scale, this sentence now reads:

*“Scores are entered into the Gold-MSI General Musical Sophistication scoring template to obtain a normalised value for each item, and these values are summed to provide an overall general musical sophistication score for each participant.”*

**Line 258: For consistency with the latter part of the sentence, “underlined” should be inserted after “second”.**
We have added ‘underlined’ to this sentence.

**Line 297: A space should be inserted after “properties”.**

This space has been added.

**Line 304: “can” should be inserted before “draw” or “draw” should be changed the “draws”. The former would be preferable.**

This has been amended with the former request (we have changed the ‘can’ to ‘may’ following your comment below).

**Lines 304-312: But is imagery really needed for the voice task? The voice task is intended to be an analogue of facial recognition, but the authors already admit that “the role of imagery in facial recognition remains unclear” (lines 281-282). I’m not suggesting that imagery can’t be used in the voice task; it might be that at least some participants used imagery at least some of the time. What I am suggesting is that it isn’t clear that imagery is required for the voice task, either for the aphantasia group or for the control group. Perhaps voices might be characterized based on descriptions of some features rather than imagery per se.**

We agree with you that we cannot be sure that imagery is required to perform the voice task. As we stated previously, we used this task because we believed that imagery could be beneficial to task performance and because it is analogous to facial recognition, a domain that has been the subject of continuing research in aphantasia (Zeman et al., 2010; Dance et al., 2023; Milton et al., 2021). We have tested for the association between the voice task and voluntary visual and auditory imagery and found no evidence of a relationship. We report this and acknowledge in the discussion that imagery might not be necessary to do this task. We have now also added an additional sentence:

“However, we did not find evidence that performance on this task was correlated with auditory imagery self-report and as such it may not have been an effective task for measuring auditory imagery ability. This may be because participants were able to identify and use acoustic features as verbal labels to differentiate between speakers without needing to generate an auditory image.”

**Lines 337-338: Why were different CV syllables used for make speakers and for female speakers? In other words, why wasn’t the same set of two (or four) CV syllables used for both male speakers and female speakers?**

We wanted to introduce different sets of CV syllables to provide variety in speaker characteristics and phonetic content over which vocal characteristics were expressed. We did not have an apriori hypothesis that the gender of the speaker would influence ease of imagery and so there was no need to balance the phonetic content across the speakers.

**Line 342: Maybe I just missed it, but what is a Bamford-Kowal-Bench sentence? How does it differ from other types of sentences? I appreciate that the authors added a reference, but this should still be clarified in the text.**

We have added an example of a Bamford-Kowal-Bench (BKB) sentence in the manuscript. BKB sentences are short 4-6 word sentences, such as “the clown had a funny face”, “they ate the lemon jelly”. BKB sentences are designed to assess speech recognition of children who are hard of hearing. For your interest, the full list of BKB sentences can be found here: <https://www.ssc.education.ed.ac.uk/courses/deaf/aud2bkb.html>. We have added detail explaining that they are short sentences with a simple syntax and vocabulary:

*“These are short 4-6 word sentences (e.g. “they ate the lemon jelly”) that have a simple syntax and vocabulary (58)”*

**Line 357: I’m not clear what “presented one by one in a fixed order to reduce confusion” means. Wouldn’t the pairs be presented one-at-a-time anyway? How does presenting a fixed order reduce confusion (e.g., were not the male voices clearly distinguishable from the female voices)? Could this fixed order supply or allow additional cues that might make imagery more or less necessary or useful?**

This is a confusion that has arisen from removing the supplementary materials. We have know re-introduced this sentence which was originally in the supplementary materials into the methods section:

*" The data from a third pair of speakers was collected but participants struggled to categorise the morphed continua into distinct vocal identities and so these data were not analysed further.”*

**Line 361: “each participant was tested one-by-one” should either be “participants were tested one-by-one” or “each participant was tested individually”. “each” implies a single participant, and you can’t have a single participant tested “one-by-one”.**

We have changed this to ‘Each participant was tested individually”.

**Line 362: The comma after “BAIS” should be deleted (or if not, then I don’t understand the sentence).**

This comma has been removed.

**Line 374-375: I’d insert a comma after “met” and delete the comma after “conducted”.**

This has been amended.

**Line 378-381: I still don’t think the authors need to list all the BF levels here. Listing interpretations for all BF values is like mentioning that p values greater than .05 won’t be considered significant in the presentations of the Mann-Whitney U tests or ANOVAs. Giving the interpretation of the BF that was actually obtained is sufficient. I leave this up to the action editor.**

Given that this is only takes up a few lines within the manuscript, we would prefer to keep it in and defer to the action editor. We argue that many readers are not familiar with Bayes-Factors and this additional information is useful and does not take up much space. .

**Line 393: “data… was”. Unless the authors are referring to the android in Star Trek, “data” is a plural term, and so “data… were” is correct (the singular form is “datum”).**

This has been changed.

**Lines 437-442: This was confusing. The authors state that the analysis was carried out using d prime values (line 438), but the results were reported in terms of accuracy (line 441). What was the dependent variable? D prime? Or accuracy?**

We have made it clearer that we are referring to d-prime values rather than accuracy (e.g. % or proportion correct). We no longer refer to accuracy to make sure that this is fully clear to readers.

**Lines 470-471: “performance on” should be inserted before both instances of “the pitch task”, as the correlation with imagery does not involve the task per se, but rather involves performance on the task.**

This has been amended.

**Line 487: Box and Cox (1964) is still not in the References section.**

Apologies for this formatting error, this now appears in the reference section.

**Lines 499-501: Whether the effect of participant group was significant or not should probably be mentioned, as well.**

We have added a sentence to the summary of this paragraph to make it clear that we are referring to imagery effects specifically.

**Line 517: If the authors wish to conform to APA style, then abbreviations such as “e.g.,” and “i.e.,” are used only within parenthetical expression. Within the main text, these should be spelled out (e.g., “for example”, “that is”, etc.)**

We have re-formatted in Vancouver style to conform to the journals default referencing style.

**Line 525: Although the readers question of “95th what?” is answered by the end of the sentence, just seeing “above the 95th” does seem a little odd.**

We have edited this sentence so that the 95^th^ is in reference to the winsorized values, and this reads:

*“…recoding all winsorized values above the 95th to the value attained at the 95% percentile”*

**Line 543: How was the combining of VVIQ and BAIS-V into a composite score done? Were the individual scores added, averaged, or was some other method of combination used?**

The composite scores were created by calculating the mean of z-scored values for each task and then averaging the z scores. The explanation is explained once earlier in the manuscript. There is a second reference to this process later on in the results (this time for the voice task) which we think this is referring to. We have now added the following text so that it is clear, when we reference it this second time:

*“There was no evidence of a relationship between auditory or visual imagery self-report and performance on the voice task alone or when the imagery measures were combined in a composite score by z-scoring and then averaging them”*

**Line 546: “BAISV” has been hyphenated previously in the manuscript (e.g., lines 393, 406, 411, 423-426, 541, etc.), and so should be hyphenated here for consistency.**

This has been amended.

**Line 573: Well, the tasks might not actually require auditory imagery if the objective performance on those tasks by people who claim to not experience auditory imagery does not differ from the objective performance by people who do claim to experience auditory imagery. Just because imagery might offer one strategy to complete a task does not mean that participants necessarily use that strategy (e.g., see Zatorre & Halpern’s, 2005, and Hubbard’s, 2010, argument why simply observing a given pattern of responses when participants are instructed to use imagery does not establish that those participants did in fact use imagery; see also Hubbard’s, 2018, discussion of representational ambiguity). Claiming that the tasks required auditory imagery is probably too strong, and it would be much more prudent to weaken this.**

We have softened this sentence, and this reads:

*“The current study compared the performance of individuals with aphantasia to participants with typical imagery on behavioural tasks that we thought would require auditory imagery.”*

**Lines 610-611: Perhaps this can be elaborated?**

We have added this text:

*“However, we investigated this empirically by testing to see if there was evidence that the distribution of auditory imagery scores in the aphantasic participants differed from a unimodal distribution (e.g., whether it reflected a sub-group with good auditory imagery and another with poor auditory imagery) and did not find evidence to support this.”*

**Lines 631-632: But the differences are evident. Perhaps the authors intended to say that the reasons for the differences, rather than the differences per se, are not evident?**

We have changed the wording in this sentence to the following:

*“There may be several reasons as to why the differences are not evident in the current study.”*

**Line 639: There is an extra opening parenthesis on this line.**
This has been removed.

**Lines 650-651: Plack et al. and Yuskaitis et al. are cited in the main text but are not listed in the References section.**

Apologies for the formatting error, these now appear in the reference list.

**Lines 658-660: “suggest” should be “suggests”. Also, an example of such evidence (even if parenthetical) would be helpful to readers.**

We have amended the word ‘suggest’ to ‘suggests’ and added the following sentence as an example of this evidence:

*“For instance, individuals with amusia have difficulties discriminating changes and pitch and perform worse on spatial tasks such as mental rotation compared to neurotypical individuals (Douglas & Bilkey, 2007; Tao et al., 2015).”*

**Line 663: What is “the equivalent”? Equivalent to what?**

We have clarified this as a ‘spatial equivalent’.

**Line 667: “, thus” should be “; thus,”**

This has been changed.

**Lines 687-689: “make the strong assumption that imagery ability in aphantasia can be upregulated by attention or similar factors”. Why? Maybe such participants used a non-imagery strategy. Just because an imagery strategy is one possibly strategy does not mean that individuals necessarily use that strategy.**

We agree, we think primarily aphantasic participants used non-imagery strategies in the tasks, and we have adjusted this sentence accordingly. This now reads as follows:

“*Given that this task explicitly asked aphantasic participants to generate auditory imagery, it may have been subject to ‘demand characteristics’. However, one might assume that such a bias would lead to reduced rather than increased performance in the aphantasic group. Given that performance was equivalent between the groups, it may that participants with aphantasia drew upon alternative non-imagery-based strategies to complete the tasks”*

**Line 690: “was less susceptible” seem a bit strong. I would be more cautious and say “was potentially less susceptible”. Also, after “characteristics, the sentence should be continued stating why such a claim is being made (i.e., “… characteristics’, because…).**

We have included the word potentially and reminded the reader as to why our task was less susceptible to demand characteristics, this reads:

*“Our voice task was potentially less susceptible to ‘demand characteristics’ because we did not explicitly instruct participants to engage in auditory imagery during the task.”*

**Line 723: “Until this is achieved… highly heterogenous”. Well, participants could still be highly heterogeneous even after a uniform diagnostic criterion is developed.]**

Yes, we agree, we have removed this sentence from the manuscript.
