# Supplementary material for: No clear evidence of a difference between individuals who self-report an absence of auditory imagery and typical imagers on auditory imagery tasks
Source: PLoS One. 2024 Apr 3;19(4):e0300219. doi: 10.1371/journal.pone.0300219 (PMC10990234; doi:10.1371/journal.pone.0300219)
Supplement: Attachment — Submitted filename: R2R_Revision3_PLOS_audImagery.docx [file pone.0300219.s003.docx]

Please use the space provided to explain your answers to the questions above. You may also include additional comments for the author, including concerns about dual publication, research ethics, or publication ethics. (Please upload your review as an attachment if it exceeds 20,000 characters)

Reviewer #1: All my comments have been addressed.

Reviewer #2: General Issues:

**Headings. I have a concern with the heading levels. In a single experiment paper, “Methods”, “Results”, and “Discussion” are the highest level headings. Subheadings such as “Participants”, “Stimuli” in the Methods section and subheadings for different tasks or analyses in the Results section should be at a lower heading level. However, all the headings in the main text appear to be at the same level.**

We are sure that further formatting of the manuscript will be undertaken by the journals production team. However, to ensure readability for the reviewer, we have adjusted the headings so that the subheadings are in a smaller font than the ‘main’ headings.

**Gold-MSI. Some parts of the description of the Gold-MSI (lines 219-232) are still confusing to me. Based on the sentence structure, I would normally interpret “self-reported singing ability and sophisticated emotional engagement” as two items, but the lack of a comma after “ability” could be read as suggesting that these might be different aspects of a single item. When I count the items on lines 220-224, I got 5 dimensions only if I consider self-reported singing ability and sophisticated emotional engagement as separate items (but readers shouldn’t have to count to resolve an ambiguity).**

To ensure utter clarity and to remove any confusion, we have edited this section and we have numbered each dimension to clearly illustrate to the reader, the 5 dimensions.

**Furthermore, I’m not entirely clear how “sophisticated emotional engagement" differs from general musical sophistication (although I admit this might just reflect a poor choice of names for the dimensions and not due to the current authors).**

These are the names of the original dimensions, with the “general musical sophistication” scale including items from all 5 of the dimensions – unfortunately we cannot comment further on the naming convention for these scales.

**Lines 226-227 implies there are 18 questions, but it isn’t clear whether this is the total number of questions in the survey or the number of questions on that dimension (and if the former, how many questions are there on each of the 5 dimensions?).**

There are a total of 18 questions. This is now clarified in the manuscript on Page 10.


Specific Issues:

**Line 37: “beyond” has connotations regarding spatial localization, and so “other than” or “in addition to” might be better.**

This has been changed to ‘other than’ on Page 2.

**Lines 58-60: I would consider reversing the order of these first two sentences, as it makes more intuitive sense (to me, at least) to first describe a general phenomenon (imagery) before describing a limitation to that phenomenon (aphantasia) than to first describe a limitation to a general phenomenon (aphantasia) and then describe the general phenomenon (imagery).**

We have reversed the order of these sentences on Page 3.

**Line 61: A space should be inserted after “documented”.**

This has been added on Page 3.

**Line 83: As “shown” was used on line 81 prior to the list, “show” should be deleted on line 83.**

This has been deleted on Page 4.

**Line 85: “imagery behavior” is potentially confusing, as imagery is generally considered internal and not observable by others, whereas behavior is generally considered external and observable by others. I wound delete “behavior”.**

We have deleted the word ‘behaviour’ from this sentence on Page 4.

**Line 95: Although grouping all the references for a series of claims at the end of a sentence is permissible, I (and probably other readers) find it more useful if a citation is given after each claim (as the authors did on lines 82-84), and I would suggest they place the relevant citation after each claim on lines 92-95 (and elsewhere in manuscript as relevant) unless of course each of the references supports each of the claims (in which case placing all the references at the end of the sentence is better).**

To avoid repetition of citations after each claim, we would like to keep the citations as a group. Indeed, other readers (including the authors) prefer a less cluttered presentation by placing all references together at the end.

**Line 102: The comma before “however” should probably be a semi-colon.**

This has been changed on Page 4.

**Line 108: I think this might be clearer if “else” is inserted before “draw”.**

This has been added on Page 5.

**Line 136: As I noted in my previous review, several style guides suggest that it is not good style to begin a paragraph with “However”. I would delete “However,”**

We have replaced ‘However’ with ‘Nevertheless’ on Page 6.

**Line 138: Commas should be inserted before and after “respectively”.**

This has been added on Page 6.

**Line 160: I would consider inserting “then” before “they would”.**

This has been added on Page 7.


**Line 171: I would suggest changing “the” to “that”.**

This has been changed on Page 7.

**Lines 175-176: “in previous research studies in this area that were collected during this time” is confusing. It isn’t clear whether “this time” refers to when the previous studies were done or to the time of the current study. Moreover, how could previous research be carried out during the current time?**

We have clarified that we are referring to the time frame of September 2019- March 2020 on Page 8.

**Line 179: I’m not sure this comma is needed.**

This comma has been removed on Page 8.

**Lines 180-182: Even if the cutoff was used and described in a previous study, readers shouldn’t have to go search for a previous document for an important piece of methodological information. The cutoff should be stated in the current manuscript (and of course, it is fine to state that the same cutoff was used in previous studies).**

We have included the cut-offs in the second sentence, and signposted this so that it is explicitly clear what these cut-offs are on Page 8.

**Line 248: I would add “each” after “20 trials”**

This has been added on Page 11.

**Lines 309, 680, 685, etc.: It isn’t necessary to use quote marks around “demand characteristics”, as the idea of demand characteristics (and the need to control for demand characteristics) has a long history in psychological research, and especially in the study of imagery (see reviews of auditory imagery such as Hubbard, 2010, Psych Bull; 2018, AP&C).**

These have been removed throughout the manuscript.

**Lines 312-356: This paragraph is too long and needs to be broken into 2-3 smaller paragraphs (which might necessitate some reorganization).**

This paragraph has been broken down to three paragraphs on Page 13 and Page 14.

**Lines 326-328: How does the “number of continua” in line 326 relate to the “4 continua” in line 328? If they are the same, then perhaps these two sentences can be consolidated.**

We have consolidated this into one sentence, which reads as follows on Page 14:

*“We used 4 continua to ensure that multiple acoustic features would contribute to voice identification and provide a variety in speaker characteristics and phonetic content over which vocal characteristics were expressed.*”

**Line 330: Perhaps “between group” should be “between-group”.**

This has been changed on Page 14.

**Line 335: A space needs to be inserted before “Prior”.**

This is now a new paragraph.

**Line 348: I might add a comma after “crucially”.**

This has been added on Page 15.

**Lines 360-362: As I noted in my previous review, several style guides recommend use of the Oxford comma (i.e., a comma after the penultimate item in a list) to maximize clarity. Accordingly, there should be a comma inserted after “Gold-MSI” and after “pitch task”. A similar lack of an Oxford comma contributed to the confusion noted in General Issue B).**

We have made this change although as the reviewer notes this is a style preference.

**Line 374: “that” should be deleted.**

This has been deleted.

**Lines 375-378: I still don’t think it’s necessary to report how any possible BF would be interpreted, but the authors clearly disagree. Nonetheless, this is a minor issue, and I won’t insist on it, but will point out that other readers might find it odd.**

Thanks, but we do think this is a worthwhile and an important addition.

**Lines 390-395: I presume the change from full to left justification for this paragraph is a typo, but it should be corrected, nonetheless.**

This has been adjusted.

**Lines 408-409: Shouldn’t “vividness of” be inserted after “between” and also before “auditory imagery”? After all, the two scales being used (VVIQ, BAIS-V) focus on vividness and not on other characteristics of the image (and previously noted, Lacey and Lawson [2013] have criticized use of “vividness” as a general evaluation of imagery, as it does not consider several other aspects of imagery [generation, manipulation, control, etc.]).**

“Vividness of” has been added twice to this sentence, which reads as follows on Page 17:

*“We further investigated the association between self-reported vividness of visual imagery (measured by the VVIQ) and vividness of auditory imagery (measured by the BAIS-V).”*

**Lines 410-412: It seems rather odd that something that shouldn’t exist (i.e., imagery in aphantasia) should be correlated with anything.**

Conceptually one could argue this, however, the aim of this analysis is to inform the reader that low vividness as represented by scores on one questionnaire for one sensory modality (i.e. vision) correlates with low vividness scores in the second sensory modality (i.e. audition).

**Lines 553-570: This paragraph is the final paragraph under the “Voice task” subheading in the Results section, but it doesn’t seem to really fit under that subheading. Perhaps a new subheading should be inserted before line 553 (e.g., perhaps something like “Further Comparison of Aphantasia and Control Groups”)? Alternatively, maybe this paragraph could be footnoted at (or added to) line 402 when the result of the initial dip test was reported?**

We have added the heading “Further Comparison of Aphantasia and Control Groups” to this section on Page 24.

**Line 605: I’m not sure the comma after “neglect” is correct. Actually, I would change this to “…conditions (e.g., hemi-spatial neglect), can…”.**

This has been edited on Page 26.

**Line 609: I would delete “However,”.**

This has been removed on Page 27.

**Line 688: Participants completed the VVIQ and BAIS questionnaires just before they did the pitch task and the voice task. It seems possible (at least in principle) that the focus on imagery in those questionnaires might have suggested to participants that imagery was an important part of the experiment. While it is good that imagery was not mentioned in the instructions to the voice task, that in itself (in light participants filling out the VVIQ and the BAIS just before completing the pitch task and the voice task) is not sufficient to rule out demand characteristics. This should also be addressed.**

We think this is unlikely, however, we have added the following sentence to the discussion on Page 29 to address this possibility:

*“Further, all questionnaires were undertaken prior to undertaking the task, which may have also suggested to participants that imagery was necessary for task performance.”*

**References: There are some inconsistences across the References section (e.g., some article titles capitalize the first letter of all of the “important" words, whereas other article title capitalize only the first letter of the first word; some journal names are abbreviated, but other journal names are spelled out in full; etc.).**

This is now corrected.

*** end of review
